# Adding Up to ADHD: Effects of Early Exposures

**Published:** 2006-12

**Authors:** Tanya Tillett

Many studies have documented health risks of childhood exposures to lead and tobacco smoke. Both exposures have been implicated in the development of attention deficit/hyperactivity disorder (ADHD) in children. A team of researchers now confirms links between both neurotoxicants and ADHD development **[*EHP* 114:1904–1909; Braun et al.]**.

ADHD, one of the most common childhood disorders, may affect up to 8% of children, costing society an estimated $9.2 billion per year. However, the mechanisms for the development of the disorder are unclear. Previous research has implicated prenatal tobacco smoke exposure in its development, but the relative contribution of this exposure remains uncertain, and to date there have been no convincing studies linking lead exposure with diagnosis of ADHD.

The researchers analyzed data collected from 3,879 children participating in the National Health and Nutrition Examination Survey. They assessed ADHD in children aged 4–15 years based on parental reports of diagnosis by a health professional and the use of medication for ADHD. They also used parental reports to estimate children’s pre- and postnatal exposure to tobacco smoke, and analyzed blood samples to determine lead concentration. The research team then used logistic regression analysis to identify predictors of ADHD.

Children exposed prenatally to tobacco smoke were 2.5 times more likely to develop ADHD than unexposed children, and those with a blood lead concentration greater than 2 μg/dL had were 4 times more likely than children with the lowest blood lead concentrations. Girls exposed prenatally to tobacco smoke were 4.6 times more likely than unexposed girls to develop ADHD, and exposed boys were twice as likely as unexposed boys. Based on these results, the researchers estimated that about 1 in 3 cases of ADHD could be attributed to either prenatal tobacco smoke exposure or childhood lead exposure.

The team acknowledges several limitations to this study, including recall bias and the inability to adjust for certain potential confounders. However, they note that their findings confirm the previously observed association of prenatal tobacco smoke exposure and ADHD as well as concern about whether low-level childhood lead exposure also is linked. This evidence reinforces the need for strengthened public health efforts aimed at reducing the occurrence of these exposures.

## Figures and Tables

**Figure:**
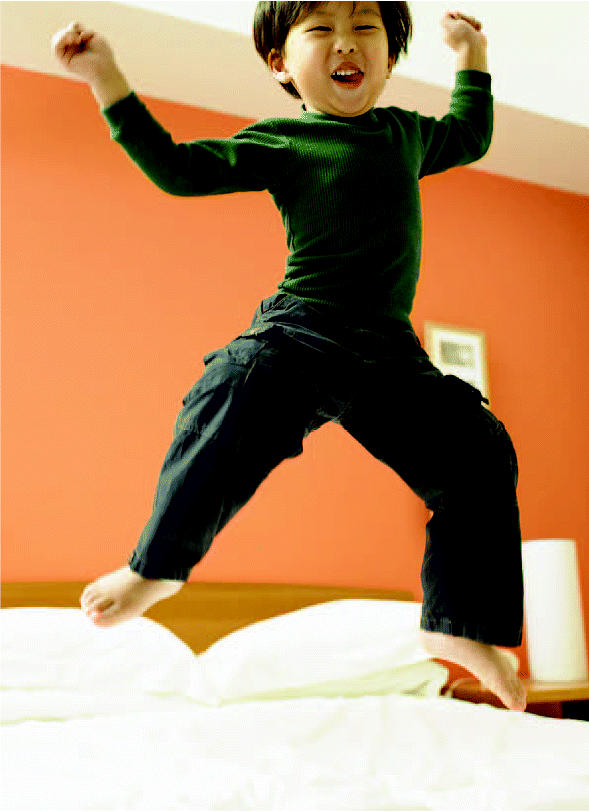
Exciting data New findings shed light on links between ADHD and early exposure to lead or tobacco smoke.

